# Until when will we grant specialist titles to doctors without medical residency?

**DOI:** 10.1590/0100-6991e-20243782-en

**Published:** 2024-09-09

**Authors:** FRANCISCO ARSEGO DE OLIVEIRA

**Affiliations:** 1 - Universidade Federal do Rio Grande do Sul, Departamento de Medicina Social - Porto Alegre - RS - Brasil

**Keywords:** Internship and Residency, Education, Medical, Specialization, Internato e Residência, Educação Médica, Especialização

## Abstract

This is a letter to the editor praising the editorial published in the Revista do Colégio Brasileiro de Cirurgiões which details the process of granting specialist titles in the field of surgery. At the same time, however, the issuance of these titles to doctors who have not completed medical residency is questioned.

## RESUMO

Trata-se de uma carta ao editor elogiando o editorial publicado na Revista do Colégio Brasileiro de Cirurgiões que detalha o processo de concessão de títulos de especialista na área da cirurgia. Ao mesmo tempo, contudo, questiona a emissão desses títulos para médicos que não concluíram residência médica.

I would like to congratulate the Journal of the Brazilian College of Surgeons (CBC) for the excellent editorial that addresses the issue of the granting of the title of specialist by medical societies^1^.

There is no doubt that scientific societies have a key role in confirming the quality of their medical specialists. Among other factors, this is what ensures greater security in the care of the population, which has the right to the best levels of health care in our country. I therefore praise the scope and technical rigor assumed by the CBC for this type of certification. 

The authors’ rationale for this is solid, based on the concepts of competence and professionalism, themes that are very dear to those who work directly with medical evaluation and training at its various levels. This is even more relevant when we refer to professionals from whom a practice of excellence is expected. 

However, the editorial ends up raising other equally important discussions, especially about the terminality of the medical course and the formation of the professional identity of doctors.

This debate is not new^2^ ^,^ ^3^. In Brazil, this issue has been discussed for more than 40 years, but always in a marginal way and, perhaps for this reason, we have advanced so little in this aspect.

International experience shows us that “terminality” has been resolved for a long time, to the point that most countries do not allow full and automatic professional practice immediately after graduation from medical school. There are usual requirements for a proficiency exam or a period of work with some type of supervision until a level of competence considered safe for independent practice is reached^4^ ^,^ ^5^. 

This is a natural consequence in the face of the increasing complexity of health care around the world and has required corresponding academic preparedness^6^. 

From this perspective, the ideal model for preparing physicians for a safe entry into the labor market is residency, still considered the gold standard in the training of specialist physicians.

It should be noted here that what defines a specialist, in addition to knowledge, skills, and attitudes, is “being” a specialist^7^. The process of professional identity formation is complex and intense, where the physician progressively incorporates, in an indelible way, the professional identity of the group to which she/he will belong. This occurs through work and remarkably close interaction with more experienced physicians in the area (preceptors), with their peers, and in well-structured services^8^. In short, this is what medical residency itself has offered for more than a hundred years and what makes it so respected. The specialist is, therefore, a professional who assumes certain characteristics of the field of knowledge and who claims an identity and the values specific to the group. This, therefore, is a concept that goes beyond the domain of skills and professionalism. 

I am aware of the enormous difficulties of the country, of regional inequalities, of the need to provide for underserved areas, and of the always insufficient budgets for health at the three levels of government. But even in this context, there are two closely related questions that need to be urgently answered: (a) Until when will we issue specialist titles to doctors without residency?; and (b) Until when will we allow a newly graduated doctor to work without any kind of supervision?

Unfortunately, we have witnessed newly graduated doctors inserted in “internships” or precarious courses, with weak regulations - especially in relation to the criteria for entry and evaluation of competencies -, with supervision that is not always adequate, a questionable theoretical part (if it even exists), and only partial dedication to the area chosen during their “training”. They are doctors who fight for a minimally decent survival by doing shifts in emergency services, walk-in clinics, or even in hospital admissions in sectors other than their professional choice, while waiting for the seal of their desired specialty to be called a “specialist”, and can, consequently, have the Specialty Qualification Registration (RQE) in the Regional Medical Councils. 

This double path to titling must end. Titles by scientific societies should be granted - with strict criteria, such as those adopted by the CBC - only to specialist physicians trained through residency programs formally accredited by the National Commission for Medical Residency (CNRM). It is evident that, for this to occur, there is a concomitant imperative to, on the one hand, expand the quantity and quality of residency programs in all specialties (and according to the needs of the country) and, on the other, ensure an adequate amount for the residency grant.

With this, we return to the issue of the terminality of the medical course, inseparable from the discussion about undergraduate and postgraduate/residency. As good as the National Curriculum Guidelines (DCNs) are, there is a need to more strongly include the concept that medical education is a continuous process, marked by periodic evaluation and certification stages, which should begin at the undergraduate level and continue for the necessary time during the professional activity. Only this way will it be possible to guarantee the standard of quality of care that Brazilian society demands and is entitled to. I understand, finally, that this must be a joint effort of the State and the whole society.

## References

[1] Pereira GA, Colleoni R, Silva LE, Bahten LCV, Fernandes CE, Portari-Filho PE (2024). Por que as Sociedades Médicas devem cada vez mais cuidar de suas provas de Título de Especialista e porque os profissionais médicos devem obtê-lo. Rev Colégio Bras Cir.

[2] Gonçalves EL (1986). A terminalidade do curso de graduação em medicina risco, problemas e soluções. R Bras Educ Med.

[3] Rocha JSY (1983). A crise da terminalidade da educação médica no Brasil. Rev Medicina HCFMRP-USP e CARL.

[4] Wijnen-Meijer M, Burdick W, Alofs L, Burgers C, ten Cate O (2013). Stages and transitions in medical education around the world Clarifying structures and terminology. Medical Teacher.

[5] Aftab W, Khan M, Rego S, Chavan N, Rahman-Shepherd A, Sharma I (2021). Variations in regulations to control standards for training and licensing of physicians a multi-country comparison. Hum Resour Health.

[6] Plsek PE, Greenhalgh T (2001). Complexity science The challenge of complexity in health care. BMJ.

[7] Cruess RL, Cruess SR, Steinert Y (2016). Amending Miller's Pyramid to Include Professional Identity Formation. Acad Med.

[8] Ronzani TM, Ribeiro MS (2003). Identidade e Formação Profissional dos Médicos. Rev Bras Educ Médica.

